# Infusing high-impact practices in undergraduate public health curricula: Models, lessons learned, and administrative considerations from two public universities

**DOI:** 10.3389/fpubh.2022.958184

**Published:** 2022-09-20

**Authors:** Sarahmona M. Przybyla, Sarah E. Cprek, Marc T. Kiviniemi

**Affiliations:** ^1^Department of Community Health and Health Behavior, School of Public Health and Health Professions, University at Buffalo, Buffalo, NY, United States; ^2^Department of Health, Behavior and Society, College of Public Health, University of Kentucky, Lexington, KY, United States

**Keywords:** high impact practices (HIPs), undergraduate public health, curriculum design, learning outcomes, public health education

## Abstract

High-impact practices (HIPs) can improve the rigor, quality, and outcomes of undergraduate education. Several high-impact practices are explicitly woven into the overarching goals, learning objectives, and curricular competencies for undergraduate public health degree programs, while others are natural fits. However, capitalizing on the value of HIPs for public health undergraduates requires a conscious effort in the process of curriculum design, course delivery, and administration of these programs. In this paper, we discuss both conceptual approaches and practical steps involved in the integration of HIPs in curriculum design and implementation. We discuss two exemplars of undergraduate programs that incorporate high-impact practices, illustrating how the same practices can be developed and implemented in different but equally effective ways across programs. We close with practical suggestions for designing or refining an undergraduate program to maximize the inclusion and effectiveness of high-impact practices.

## Introduction

An undergraduate education has moved from an elite opportunity to the standard for a majority of American young adults. In 2016, nearly 70% of high school graduates between the age 18–26 enrolled in college (1), whereas only about 5% of Americans earned a bachelor's degree in 1950 (2). Both the significant enrollment increase and the substantial shift in racial/ethnic and socioeconomic diversity of the undergraduate population has required higher education to critically assess both the goal and process of educating the majority of the country's high school graduates.

As a part of a broader initiative to provide essential student learning outcomes for higher education programs throughout the nation (3, 4), the American Association of Colleges and Universities led an initiative to identify effective teaching and learning strategies, referred to as High Impact Practices (HIPs), which support student learning and success (5). This initiative included an assessment of students who historically experience lower rates of success (e.g., retention, degree attainment) within the field of post-secondary education, specifically first-generation college students, racial/ethnic minority students, and students living in poverty (5, 6).

The HIPs commonly discussed in the literature include: common intellectual experiences; writing intensive courses; collaborative assignments and projects; undergraduate research; diversity and global learning; service and community-based learning; capstone courses and projects; first-year seminars and experiences; learning communities; ePortfolios; and internships (5–7). Use of these HIPs lead to improved student learning, increased retention, and improved engagement (6). Further, there is a significant dose-response relationship between the number of HIPs experienced by students and overall student success. Significantly, research has demonstrated that historically disadvantaged students show a greater positive academic impact from experiencing HIPs, resulting in a reduction in the achievement gap between these students and the traditionally advantaged undergraduate student population (6).

### Goals of undergraduate education in public health, high-impact practices, and liberal education learning outcomes—Integrations and opportunities

A review of the common goals of undergraduate education in public health, as articulated in the scholarship of teaching and learning literature and codified in the Council on Education in Public Health (CEPH) accreditation criteria for undergraduate public health education finds many points of commonality with the defined set of HIPs. In addition, there are commonalities between the criteria, HIPs, and general articulations of the goals of general education/liberal education programs at universities, such as the widely-used LEAP (Liberal Education and America's Promise) learning outcomes developed by the American Association of Colleges and Universities (8).

In recent years, there have been several examinations of the goals specific to undergraduate public health education. Three broad goal categories have been suggested. First, undergraduate public health education has been situated within a liberal education framework, where the predominant goal is to give students the critical thinking skills, broad general education, and content area knowledge to be able to effectively problem solve, adapt into new roles, and apply public health thinking and practices to a variety of situations (9–11). A second framing centers undergraduate public health education in a professional education framework, with the primary goal being educating students to develop the skills needed to be successful in the public health workforce at the bachelor's level or to have the foundation of training to be successful in the workforce at higher levels with additional masters or doctoral- level training (12, 13). Finally, the third goal is that the bachelor's level training in public health is also preparation for a variety of both academic graduate and professional school career paths—including but not limited to graduate-level training in public health (e.g., MPH), doctoral degrees in public health research areas, and health professions training (e.g., medical school, dental school). This goal is common to both the liberal education and professional education framing of the degree.

Common to each of these goals is both content area knowledge and a strong set of intellectual and practical skills—critical thinking, problem solving, collaboration, communication, civic engagement, and project management, just to name a few (14, 15). The goal of HIPs are to provide robust preparation and therefore the best educational outcomes for students in these skillsets (5). Thus, incorporating HIPs into public health education is central to ensuring that our students leave their undergraduate training with the skills needed to be part of the public health workforce and the educated citizenry.

Because HIPs are implemented in and have their effects on learning through specific curricula (both in general education and within specific majors), effectively infusing HIPs into undergraduate public health programs should begin with consideration of the extent to which general education, undergraduate public health major curricula, and HIPs overlap and relate to one another. Identifying connections that can be used to infuse HIPs into the public health curriculum as well as general education offerings is a key step to effectively using HIPs in undergraduate education.

In Table 1, we present a “matching” of HIPs to CEPH's required elements of the undergraduate major in public health. In addition, because undergraduate majors are situated within a broader university curriculum that includes general education, we examine the relation of the CEPH learning outcomes and HIPs to the general education outcomes specified in the LEAP program (8).

**Table 1:** Mapping of high impact practices to CEPH accreditation requirements for undergraduate public health and AACU's LEAP essential learning outcomes.

**HIPs**	**CEPH criteria (2016)**	**LEAP essential learning outcomes**
**HIPS core to UGPH curriculum and goals**		
Common intellectual experiences	UGPH curricula – General education: D9 – overall curriculum introduces students to foundations of scientific knowledge, social/behavioral sciences, statistics, humanities/fine arts	Knowledge of human cultures and the physical and natural world
Diversity and global learning	D10: the socioeconomic, behavioral, biological, environmental and other factors that impact human health and contribute to health disparities the fundamental characteristics and organizational structures of the US health system as well as the differences between systems in other countries	Personal and social responsibility, including: Civic knowledge and engagement—local and global Intercultural knowledge and competence
Capstone courses and projects	D12: All students complete a cumulative, integrative and scholarly or applied experience or inquiry project that serves as a capstone to the education experience.	Integrative and applied learning, including: Synthesis and advanced accomplishment across general and specialized studies
**HIPs naturally synergistic with UGPH**		
Writing intensive courses	D10: basic concepts of public health-specific communication, including technical and professional writing and the use of mass media and electronic technology D11: basic concepts of public health-specific communication, including technical and professional writing and the use of mass media and electronic technology	Intellectual and practical skills, including: written and oral communication
Collaborative assignments and projects	D13: networking, organizational dynamics, teamwork and leadership	Intellectual and practical skills, including: Teamwork and problem solving
Undergraduate research	the basic concepts, methods and tools of public health data collection, use and analysis and why evidence-based approaches are an essential part of public health practice	Intellectual and practical skills, including: Inquiry and analysis Critical and creative thinking
Service learning, community-based learning		Personal and social responsibility, including Civic knowledge and engagement—local and global
Internships		Personal and social responsibility, including *Anchored* through active involvement with diverse communities and real-world challenges
**HIPs with scope beyond a single academic program**
First-year seminars and experiences		
Learning communities		
ePortfolios		

There are three HIPs (common intellectual experience, diversity and global learning, and capstone experiences) that match closely to specific accreditation requirements and are therefore almost certainly part of any undergraduate public health curriculum. Given the core knowledge elements in the CEPH criteria as well as the interdisciplinary and generalist nature of public health practice, virtually all undergraduate public health curricula provide a common core. Given the emphasis on social determinants, health disparities, and cross-national health systems in the CEPH criteria, a focus on diversity and global learning is also instinctively implied. Finally, a capstone experience is specifically required in the CEPH criteria.

Next, there are a group of HIPs that we would argue are natural fits with the undergraduate public health degree and can and should be integrated into curriculum design wherever possible. Writing-intensive courses are a high impact way to meet the CEPH criteria's focus on communication and writing skills. Collaborative course assignments provide students with training in the cross-cutting competencies of networking, organizational dynamics, and teamwork/leadership. Undergraduate research experiences, in addition to preparing students for public health research careers and graduate education, address competencies in the methods and tools of public health data collection and analysis. Finally, both service-learning opportunities and internships provide ways to address learning goals for public health students.

The remaining HIPs, first-year seminars, learning communities, and ePortfolios are, by their nature more university-wide in terms of structure, oversight, and delivery. Although we would strongly encourage undergraduate public health programs to pursue involvement in these HIPs, they fall outside of the focus of this paper given that they go beyond the public health curriculum and program itself.

## Learning environment

The following section will provide concrete examples of incorporating HIPs into undergraduate public health curricula. We will explore two undergraduate public health programs, the University at Buffalo's Bachelor of Science in Public Health and the University of Kentucky's Bachelor of Public Health. For each, we will show how and where specific HIPs are incorporated into specific courses, as well as how those experiences are mindfully scaffolded across the 4-year student experience. Finally, we will discuss administrative considerations and challenges for each program.

### University at Buffalo's Bachelor of Science in Public Health program

#### Setting

With more than 32,000 students, the University at Buffalo (Buffalo) is a public research university and member of the Association of American Universities. Buffalo's School of Public Health and Health Professions (SPHHP) was awarded full CEPH accreditation in 2009 and reaccredited in June 2015. SPHHP launched its Bachelor of Science in Public Health (BSPH) program in Fall 2017.

#### Students

Students can declare the BSPH major as incoming students (both first year and transfer students) or can transfer into the program at a later point in their academic trajectory. All Buffalo undergraduates complete 60 credit hours of general education. Students typically complete the pre-major coursework during their first 2 years of undergraduate study. During this time, students focus on 60 credit hours of university-required academic preparation (UB Curriculum). The UB Curriculum requirements include classes in writing, math and natural sciences that promote quantitative reasoning, diversity, cultural competency, ethical and analytic reasoning, and enhanced communication. Students begin with the UB Seminar, a three-credit course on a topic of their choosing in the first semester of their freshmen year. The course focuses on critical thinking skills and reflective discussion. Students then complete the following: (1) Foundations (building blocks of academic inquiry with an emphasis on communication literacy, quantitative reasoning, scientific inquiry, and diversity),(2) Pathways (a series of thematically linked courses totaling 9 credits and a global pathway, totaling 9 credits), and (3) Capstone (a culminating course, typically in the last year of study, that focuses on connections across academic disciplines). Three required BSPH courses fulfill UB Curriculum requirements. BSPH students pursue an additional 60 credit hours toward the major, including 19 credits outside of public health. These external courses include chemistry, political science, statistics, psychology or sociology, and human physiology. The remaining 41 credits includes introductory coursework, upper-level coursework, nine credit hours of free elective courses, and one four-credit capstone course.

At present, there are approximately 475 BSPH students in the Undergraduate Public Health Program (UGPH). Approximately 35% identify as an underrepresented minority, 75% identify as women, and 26% are first-generation college students. Comparatively, of the overall undergraduate student population at the University at Buffalo in Fall 2021, 24% identified as an underrepresented minority while 19% were first-generation. In addition, 4% are members of the University's Honors College. The official 6-year graduation rates for the program will become available in 2024. However, among the first program cohort (*n* = 17), 100% have completed their BSPH degree.

#### Faculty

Currently, there are seven full-time clinical assistant professors who teach primarily in the UGPH program. There are also faculty in the SPHHP's Department of Community Health and Health Behavior and Department of Epidemiology and Environmental Health who also cover specific UGPH courses as well as adjunct faculty support from public health researchers and practitioners in the Western New York community.

### University of Kentucky's Bachelors of Public Health program

#### Setting

The University of Kentucky (Kentucky), a Carnegie Research I and a land grant institution, as well as the flagship university for the state, educates over 30,000 students annually. The university is a member of the Southern Association of Colleges and Schools. Kentucky's College of Public Health (CPH) was awarded full CEPH accreditation in 2005 and was last reaccredited in September 2017. CPH launched its Bachelor of Public Health (BPH) program in Fall 2014.

#### Students

The BPH is a selective admission program. Students meeting the university standard admission requirement are eligible to declare pre-BPH as their major. Transfer students meeting the program requirements of a 2.75 cumulative GPA and 3.0 program specific GPA are also eligible to declare as pre-BPH majors. Program specific pre-major courses include three public health classes: introductory public health, gerontology, and biostatistics. The final three pre-major required classes include biology, math, and medical terminology. Students typically complete the pre-major coursework during their first 2 years of undergraduate study. During this time, students also focus their study on the 30 h of university required liberal arts core (UK Core), which ensures students are introduced to foundational material across academic disciplines. The UK Core requirements include classes in mathematics, social science, humanities, arts, communication, community citizenship, and global dynamics. Five of the required pre-BPH courses fulfill UK Core requirements. This allows students who do not meet our GPA requirements for admission to still be able to use the courses completed toward another degree program. After successfully completing the pre-major courses and meeting the GPA requirements, students may apply for admission into the BPH program.

The program admits 100–175 students per academic year. Currently there are nearly 180 students in the BPH program, with an additional 110 students declared as pre-BPH majors. The program is popular among underrepresented minority and first-generation students, who make up 22 and 35% of the program student body, respectively. The student body is predominantly female, with 86% of students identifying as such. Comparatively, 17% of the Kentucky undergraduate student body identifies as an underrepresented minority, while 27% are first generation.

Once admitted to the BPH program, students complete 15 credit hours of the BPH Core coursework, followed by an additional 9 h of senior-level required courses. Students are also required to complete 18 h of public health electives. Outside of the required public health courses, students complete 9 h of social science and 6 h of natural science electives. They also complete the Kentucky liberal arts core coursework, a requirement for all Kentucky bachelor degrees. In total, the degree requires a minimum of 120 credit hours. The current 6-year graduation rate for the program is 91%, compared to the University rate of 68%.

#### Faculty

CPH has two teaching-focused Assistant Professors and one full-time Lecturer that teach exclusively in the BPH program. An additional 7 Assistant Professors, 5 Associate Professors, and 4 Professors regularly teach BPH courses. The college also partners with public health researchers and practitioners from the community to offer topic-specific courses as adjunct faculty.

## Course and curriculum design

### University at Buffalo's Bachelor of Science in Public Health program

In the design, facilitation, and implementation of the BSPH curriculum at Buffalo, courses were individually and collectively developed with an intentional purpose for active student engagement. Within the 11 HIPs outlined by Kuh (5), the BSPH program at the University at Buffalo incorporates seven practices, outlined below.

#### Common intellectual experiences

The BSPH curriculum has been designed with an intentional integrative approach instead of a siloed style (16). Rather than following a vertically organized curricular pattern, students approach the BSPH curriculum with an integrative design such that course content is introduced, reinforced, and strengthened throughout their curricular experience.

#### Writing-intensive courses

Written assignments are included in virtually every BSPH course. Across the curriculum, assignments are designed to teach the value of and skills for writing for different audiences. In addition, upper-division core coursework of includes *Models and Mechanisms for Understanding Public Health (PUB320)* and *Interventions to Improve Public Health Problems (PUB325)*. These courses, taken in sequence, iteratively review key models to explain public health problems (e.g., social ecological model, epidemiologic triad) paired with public health interventions to improve population health and wellbeing. Learning outcomes in both are achieved using a variety of written assignments. In addition, *Introduction to Public Health (PUB101)* includes several small-scale writing assignments to develop academic writing skills.

#### Collaborative assignments and projects

Within the core BSPH curriculum, coursework includes a range of group activities and assignments. For example, in *PUB320*, students participate on debate teams to apply health behavior change theories to real world public health problems, helping students develop critical thinking skills on theoretical applications for different target populations. Students learn effective team-based, problem-solving skills in *Systems and Policies for Public Health (PUB330)*. In-class learning activities include a case studies and instructor facilitated discussions where students collaborate in small teams to explore current health policy topics. Finally, students in *Health and Disease: Biological, Personal and Environmental Influences (PUB310)* complete small group, in-class activities using *TopHat*, an interactive learning experiences platform that is seamlessly integrated with the university's course management system.

#### Undergraduate research

At Buffalo, undergraduate students can take part in research opportunities for course credit, typically in their third or fourth year. Undergraduate research is not a program requirement and does not count as an elective for degree requirements, although it counts toward the minimum 120 credit hours needed for degree completion. Some UG students may take on research opportunities in either paid or volunteer capacities (not for credit) with faculty. There were 15 BSPH students enrolled in independent study or undergraduate research courses in Spring 2022.

#### Diversity/global learning

BSPH students are encouraged to pursue a wide variety of opportunities to learn with a global lens. First, students take *Global Public Health (PUB210)* to learn about the leading causes of illness, death, and disability globally and the importance of public health approaches to prevention and control of those conditions in resource-constrained settings. Students also learn about the complex interrelationships between social, environmental, structural, and political factors that affect health and wellbeing in low- and middle-income countries. While this course is one of three 200-level electives in the BSPH program, it is offered more frequently than the other 200-level electives. In the Spring 2022 graduating cohort, 81% of BSPH students had completed *PUB210*. Second, students may take part in faculty-led study abroad programs through the SPHHP Office of Global Health Initiatives. In addition, PUB330 includes content on comparative health systems and health policies outside the United States. Finally, Buffalo's general education requirements include a 9-credit “Global Pathway”. Students choose one of three tracks—global reflections, language and culture, or international experiences.

#### Community-based learning

In the BSPH curriculum, experiential learning is a common instructional strategy. Courses give students tools to not simply learn concepts but create effective solutions in their communities. Reflective writing assignments also generate solution-oriented approaches rather than simply a laundry list of public health problems and their underlying behavioral and socio-structural determinants. For example, students in *PUB325* complete a Photovoice project, completing a written assignment and oral presentation about a photograph representing their point of view regarding a public health concern in their community.

#### Capstone courses and projects

After completing all required public health coursework, BSPH students complete a four-credit capstone experience. *Modern Public Health Problems and Solutions (PUB494)* offers the opportunity to holistically integrate previous public health coursework and out-of-classroom experiences. The seminar-structured course focuses on integrating and synthesizing knowledge gained in the major's core curriculum and using that knowledge to analyze, explain, and address public health problems. Students also gain exposure to how that knowledge is applied in public health practice. The course is centered around student projects based on case studies of public health problems, culminating in a public health problem and solution written assignment with an accompanying oral presentation.

### University of Kentucky's Bachelors of Public Health program

Similar to Buffalo, the BPH program curriculum at Kentucky strategically incorporates seven of the 11 HIPs (5). A summary of how these experiences are infused within our courses is summarized below.

#### Common intellectual experiences

The Kentucky BPH is a selective admission program that incorporates a cohort model for upper division students. In the first academic year following admission into the upper division, students complete five required courses with their cohort. During their senior year, they complete three additional required courses with this group. Cohorts include between 100 and 175 students. Each required course has an enrollment cap of 50 students to help support active learning and collaborative experiences with classes. These upper division classes are restricted to students admitted to the program, so students have the opportunity in these classes to build relationships with members of their cohort.

Since we restrict class size to 50 students, it requires us to offer multiple sections of each required class. This approach means that students do not take every class with the same group of students, but across the final 2 years in the program students will have the majority of classes with a group of students from their cohort. As the program has grown to require multiple sections of each course, we have become mindful of needing to provide additional support to strengthen the relationships within the cohort outside of the traditional classroom setting. The College is currently working to further support this relationship building between members of a cohort by hosting social events throughout the academic year. We are also piloting study groups among pre-BPH student cohorts to build out those relationships earlier in their academic career.

#### Writing-intensive courses

Writing is emphasized throughout the Kentucky BPH coursework. The program includes three writing-intensive courses that utilize a Writing Across the Curriculum/Writing Within the Discipline (WAC/WWD) framework to strengthen both academic writing skills and knowledge retention of student learning outcomes (17, 18). Our *Introduction to Public Health (CPH201)* course, which is taken as a pre-major, introduces students to the WAC/WWD model which divides large written assignments into multiple small-scale/low-stakes writing assignments throughout the semester. Students are then provided an opportunity to revise, combine, and resubmit these assignments into a final written product. This pedagogical technique is reinforced in our *Foundations of Health Behavior (CPH44) course*, where over the semester students produce an 18-page, theory-informed health behavior intervention program. Finally, in *Capstone (CPH470)* students apply their knowledge by producing a 25-page case-study assessing a specific community in relation to a public health problem and explore the fit of a particular evidence-based intervention or policy using a systems-thinking approach.

#### Collaborative assignments and projects

Collaborative projects are emphasized in several courses. While there is some group work is most courses, two courses incorporate a major group assignment as a key course component. In *Fundamentals of Environmental Health (CPH320)* students work in groups to produce a digital documentary investigating a chosen environmental hazard. In *Health Analytics I (CPH330)*, students again work collaboratively to produce a research poster on a selected public health topic. This involves investigating literature on the topic, analyzing statistical relationships, and presenting their findings to their peers and program faculty as a final assignment.

#### Undergraduate research

The Kentucky BPH offers upper-division students the opportunity to apply for undergraduate research placement. While undergraduate research is not a program requirement, it can be used to fulfill up to six of the required 18 credit hours of public health electives. An undergraduate research director promotes the experience to all students in the program and faculty in the college. Interested students indicate their research interests and preferred level of independent work. Students must complete human subjects research certification to be eligible for placement.

The college historically has had high rates of faculty mentorship each year. Over the previous 3 years, 30 faculty have worked directly with 95 students within the program. The university and college have provided financial support to students to further support undergraduate research projects, including competitive paid research fellowships to students who plan to continue their research following the completion of an independent study experience. Students who receive the CPH research fellowship are required to present their research at one of two research events hosted at the university each spring.

#### Diversity/global learning

The BPH program requires *A Sick World: Global Public Health in the Early 21st Century* (CPH476G) to ensure all students receive instruction on the global prospective of public health. In addition, the program offers several global-focused electives and study abroad opportunities that can be used to fulfill a portion of the required 18 credit hours of public health elective coursework. The Kentucky BPH program has also infused content on health equity among diverse populations throughout several required courses to ensure students have an opportunity to fully engage with and understand this critical concept. Students are introduced to the topic in *CPH201* and*: Aging in Today's World (GRN250)*. The concepts are reinforced across several core courses including *Foundations of Environmental Health (CPH320), Health Systems and Policy (CPH350)*, and *Foundations of Health Behavior (CPH440)*. This information is then applied throughout *Public Health Practice and Communication* (CPH455) and *Capstone (CPH470)*.

#### Community-based learning

Several courses in the BPH program incorporate community-based learning. Specifically, CPH440 and CPH470 require students to utilize solution-based approaches to community-specific situations after completing a community assessment. CPH455 requires a 20-h community volunteer experience. Several upper-division electives also include experiential or service-based learning activities.

#### Capstone courses and projects

*CPH470* is the culminating course in the major. Students are required to complete all of the public health core coursework prior to taking Capstone. The course takes a systems-thinking approach and utilizes a WAC/WWD framework, as mentioned above. Students are required to apply knowledge gained throughout the academic program in the completion of this project.

## Programmatic scaffolding

Curricular scaffolding in higher education courses and bachelor programs has been found to positively impact learning as it allows students to revisit content, tackle more complex topics, and gain independence in application of critical concepts over time (19, 20). Throughout this section we will explore how the two undergraduate public health programs have incorporated program level scaffolding across the curriculum.

### University at Buffalo's Bachelor of Science in Public Health program

**Figure 1:**
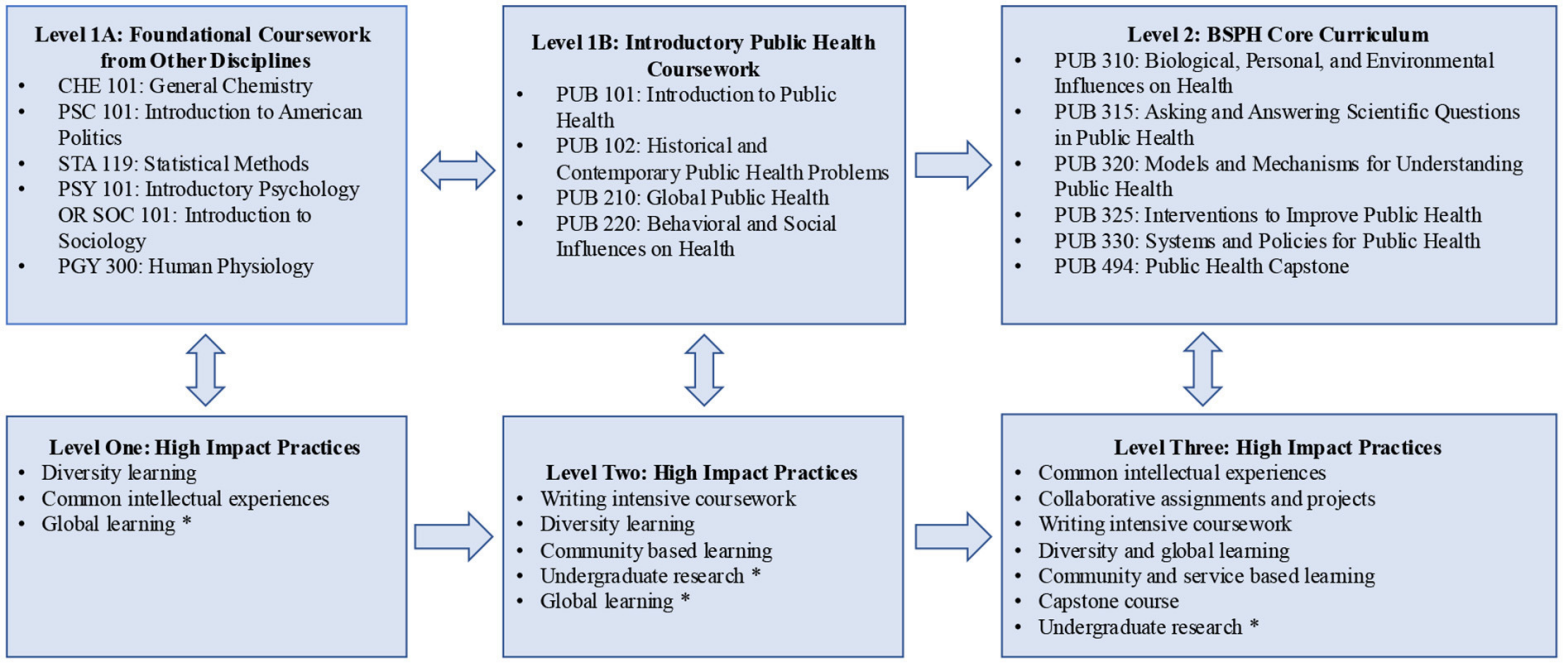
University at Buffalo: Bachelor of Public Health program scaffolding and HIPs. * Optional experience based on student selected public health electives.

The Buffalo BSPH curriculum incorporates academic scaffolding using a two-level approach which maps to HIPs in a vertical fashion. Unlike Kentucky's model, level one includes two parallel tracks (Level 1A and 1B) rather than sequential (see Figure 1). A description of the BSPH program scaffolding and related HIPs are provided below.

At Buffalo, students can enroll in foundational coursework from other disciplines (e.g., political science, chemistry) in tandem with introductory public health coursework. In fact, more students enter the BSPH from other majors than as direct entry, first-year BSPH students. Consequently, a large proportion of new BSPH students have already completed this foundational coursework prior to or in the same semester as *Introduction to Public Health (PUB101)*. One of the five required courses is *Human Physiology (PGY300)*, a four-credit course offered through the Buffalo Jacobs School of Medicine and Biomedical Sciences. One of the required courses is *Statistical Methods (STA119)*, a four-credit course offered in the Biostatistics Department. The remaining courses are offered through the Buffalo College of Arts and Sciences, including *General Chemistry (CHE101), Introduction to American Politics (PSC101)*, and a choice of either an introductory course in Psychology or Sociology. This foundational knowledge is critical as each of these courses is a pre-requisite for the Level Two courses.

*PUB101* is the first required writing intensive course in the program. In addition, *Historical and Contemporary Public Health Problems* (PUB102) focuses on diversity- and community-based learning principles from a public health perspective. Students can begin upper-division, Level 2 coursework following pre-requisite course completion in Levels 1A and B. The core curriculum is typically completed in one's junior year and includes five courses, typically completed over two semesters. In addition to common intellectual experiences, students experience writing intensive coursework, community-based learning, and collaborative projects and assignments across several of these courses. The final Level 2 component includes the four-credit capstone course, almost exclusively taken in a student's final semester. The HIPs included in the capstone are writing intensive coursework, diversity and global learning, and capstone projects.

Students are required to complete 15 h of public health electives. These are also leveled, with students required to complete a minimum of six credit hours at the 200-level and nine credits at the 300/400-level. This approach allows students to select lower- and upper-level electives that fit their interests. For example, *Introduction to Epidemiology* (PUB340) is an elective course, covering content beyond the epidemiological concepts in the integrative core courses. The 400-level electives are topic specific (e.g., Public Health Nutrition, Maternal, and Child Health) and provide students choices as they round out their coursework in their final year of study. Finally, students may also select electives that include the HIPs of diversity and global learning, and collaborative assignments and projects. Figure 1 outlines the general structure of the Buffalo BSPH academic scaffolding.

### University of Kentucky's Bachelors in Public Health program

**Figure 2:**
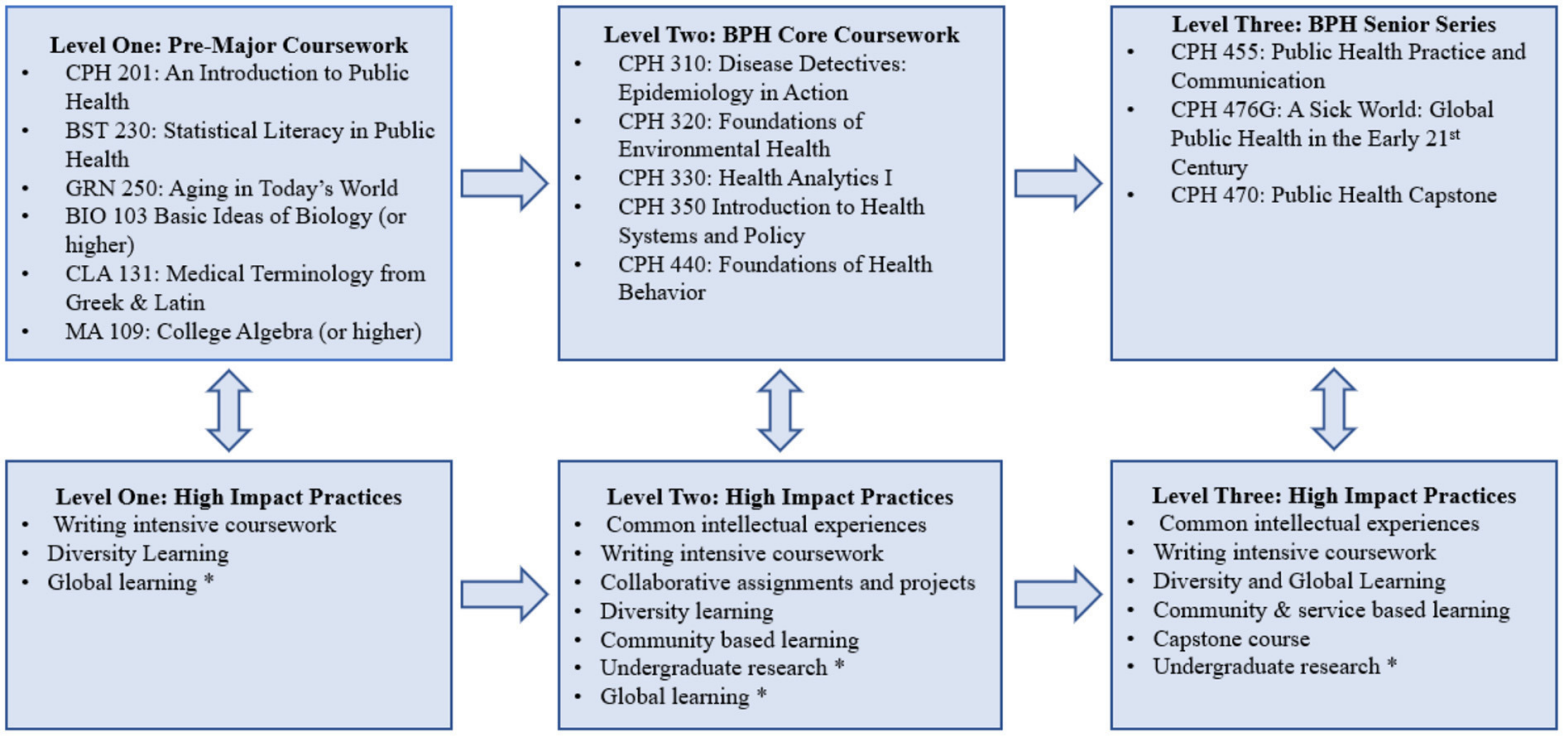
University at Kentucky: Bachelor of Public Health program scaffolding and HIPs. * Optional experience based on student selected public health electives.

The Kentucky BPH program has incorporated academic scaffolding both to increase the rigor of courses and to strengthen student learning. The academic scaffolding includes three primary levels (see Figure 2). Each includes mapped HIPs to ensure students have these experiences throughout the undergraduate program. A summary of each of the three levels and their mapped or optional HIPs are provided below.

Students must first complete six pre-major courses including an introductory course to public health, biostatistics, gerontology, biology, math, and medical terminology. Three of the required courses are offered by CPH include *An Introduction to Public Health* (CPH201) which is the first required writing intensive course in the program. Students also receive foundational knowledge for future undergraduate research experiences in *Statistical Literacy in Public Health* (BST230). Finally, in both CPH 201 and *Aging in Today's World (GRN250)*, students are introduced to concepts of health equity.

The BPH program admits students to upper division once per year, which allows for the incorporation of common intellectual experiences. Following admission, students enter the second level of the academic scaffolding in the major core coursework (i.e., epidemiology, biostatistics, environmental health, heath systems and policy, and health behavior). Students typically take these five courses over their first two semesters in the upper-division program. Beyond common intellectual experiences, students experience writing intensive coursework, diversity, community-based learning, and collaborative learning across these courses.

The final level of academic scaffolding includes three courses typically taken in the student's senior year, including a public health practice and communication course, a global health course, and the program's Capstone. The HIPs included in these three courses are writing intensive coursework, diversity and global learning, service and community-based learning, and capstone projects.

Throughout the program, students are required to complete 18 credit hours of public health electives. These are also leveled, with students required to complete a minimum of six credit hours at each of the 200, 300, and 400 levels. This approach helps to ensure students are given an opportunity to generally explore public health elective coursework early in their academic career while being required to apply higher-level concepts in more rigorous elective courses as they approach the end of their degree. The majority of the program's 300 and 400 level electives incorporate service or community learning components. Students may also select electives that include the HIPs of undergraduate research, diversity and global learning, and collaborative assignments.

The image below outlines the general structure of the Kentucky BPH academic scaffolding.

## Administration

### University at Buffalo's Bachelor of Science in Public Health program

The Buffalo School of Public Health and Health Professions established an UGPH Advisory Committee in 2014 to provide administrative structure and support for the BSPH program. This committee includes the UGPH Program Director, Director of Undergraduate Advising, and a faculty representative from each department in the School. As the program has become more established, the frequency of committee meetings has been reduced. As the UGPH program is operationally structured out of the Dean's Office in the School rather than a standalone academic department, the Dean's Office provides ongoing support for faculty recruitment and retention and faculty trainings on pedagogy and teaching effectiveness, including HIPs. Monthly UGPH faculty team meetings, led by the program director, also aid in supporting curricular planning with respect to course scaffolding and delivering high-quality, student-centered learning opportunities.

### University of Kentucky's Bachelors in Public Health program

The University of Kentucky College of Public Health developed the Undergraduate Committee (UC) in 2016 to provide administrative structure and faculty oversight for the BPH program. This committee, which includes the Director of Undergraduate Studies (DUS), the Associate Dean of Academic and Student Affairs, the Director of Undergraduate Advising, a faculty representative from each department in the college, and two student members, has been invaluable to the development and management of the program. This collaborative committee took an early lead in the assessment of HIPs in the program, and then worked together with program faculty to strengthen and streamline these components. One way this has been done is by hosting an annual summer workshop for all faculty who teach in the program. This full-day event includes a review of the program curriculum and discussions of the 4-year program structure. It has also included sessions on HIPs, which has helped to strengthen and diversify how and when students encounter these experiences.

Beyond the UC, the Kentucky BPH program has found success through a shared commitment to students and education within the college. CPH has invested in the BPH program and its students through the hiring of three teaching-intensive faculty lines which include protected time to support the BPH program. These faculty positions, which includes the DUS line, have been critical in supporting the program and its faculty. Further, the college has continued to grow and strengthen the number of support staff who work directly with undergraduate students. This financial commitment to dedicated faculty and staff administrative time has allowed for a thoughtful and strategic adoption of the scaffolded, HIPs-focused curriculum.

## Program administration considerations

Both universities have developed strong undergraduate academic programs that emphasize the incorporation of HIPs throughout their academic programs. Through thoughtful course design and collaborative planning and implementation, the students from each program will encounter seven unique HIPs prior to graduation.

Undergraduate public health program directors should consider four key points with respect to program administration and the effective utilization of HIPs. First, establishing and reinforcing a culture of openness and communication is essential. Creating shared resources across faculty members (including adjunct instructors) demonstrates the value of community. For example, the Buffalo strategy has included shared electronic resources including syllabi, in-class learning activities, and written assignments. In addition, Buffalo faculty meet monthly to discuss integrative approaches between and across coursework. Similarly, as previously mentioned, Kentucky hosts an annual full day workshop for faculty who teach in the BPH program to collaborate and strengthen the academic experience.

Second, for faculty investment in HIPs to be realized, their keen engagement across the curriculum is consequential. For example, the Buffalo approach has included a recurring rotation of full-time faculty across various courses from introductory to upper-division. Kentucky has addressed this through an investment in full-time faculty lines dedicated specifically to teaching within the BPH program, while rotating in research intensive faculty regularly.

Third, institutional priorities and contexts may influence the extent to which HIPs can be fully implemented. As such, it is important to consider flexible, nimble variations in learning and instruction particularly when circumstances and events necessitate a revision to the original course delivery mode (e.g., transitioning a course from fully in-person to a remote, synchronous method of delivery). Context can also shape decisions about the implementation of HIPs. For example, some undergraduate public health programs fully incorporate internships into their curricula. At both UK and UB, internships were considered when programs were designed. However, both institutions are situated in mid-sized cities and both already had existing, robust MPH programs with a field experience requirement when the undergraduate programs were created. Given those realities, administration and faculty at both institutions concluded that internships for undergraduates, although pedagogically beneficial, could not be feasibly implemented given the finite number of placement opportunities and the existing needs of each their respective MPH programs.

Finally, planful and systematic implementation of HIPs across an undergraduate curriculum requires equally planful administration and resourcing for the program. From an administrative perspective, both the number of faculty required and the stability with which faculty are teaching courses in the undergraduate program will need to be considered given that overall faculty/student ratios and individual class sizes can both impact the feasibility of implementing HIPs. At UB, this has been primarily accomplished through hiring non-tenure track, teaching-oriented faculty whose job responsibilities are explicitly focused on the undergraduate public health program. At UK, there are not specific faculty identified as undergraduate program faculty, but active efforts have been made to recruit faculty to teach in the undergraduate program and to work with department chairs to ensure continuity of course coverage over time.

In addition, institutional budgetary models need to be leveraged to ensure the ability to successfully implement a HIPs-infused curriculum. At institutions with a responsibility-centered management (RCM) budget model, careful consideration must be given to the balance between the fiscal realities of enrollment and class size as a revenue generator vs. the pedagogical reality that class sizes have an effect on instructor effort and other resources needed to implement HIPs successfully. At institutions with incremental or other budget models, the revenue vs. pedagogy balance may not be as explicit, but the necessity to ensure that fiscal resources for the program are sufficient is no less important. A full discussion of business models for undergraduate public health programs is well beyond the scope of this article (as well as being relatively institution-specific).

## Conclusion

Conceptual, logistical, and practical approaches to the integration of HIPs into undergraduate public health curricular design and implementation are important for faculty and administrators at schools and programs of public health who seek to develop new bachelor's degree programs or modify existing programs. Thoughtful, deliberate application and execution of such approaches aid in optimizing the value of HIPs into undergraduate education to best prepare the next generation of public health researchers and practitioners.

## Data availability statement

The original contributions presented in the study are included in the article/supplementary material, further inquiries can be directed to the corresponding author.

## Author contributions

All authors contributed to conceptualization of the manuscript topic, writing of the first draft, editing, and final approval.

## Conflict of interest

The authors declare that the research was conducted in the absence of any commercial or financial relationships that could be construed as a potential conflict of interest.

## Publisher's note

All claims expressed in this article are solely those of the authors and do not necessarily represent those of their affiliated organizations, or those of the publisher, the editors and the reviewers. Any product that may be evaluated in this article, or claim that may be made by its manufacturer, is not guaranteed or endorsed by the publisher.
